# Electroosmotic Flow of Viscoelastic Fluid in a Nanochannel Connecting Two Reservoirs

**DOI:** 10.3390/mi10110747

**Published:** 2019-10-31

**Authors:** Lanju Mei, Shizhi Qian

**Affiliations:** 1 Department of Engineering and Aviation Sciences, University of Maryland Eastern Shore, Princess Anne, MD 21853, USA; lmei@umes.edu; 2 Department of Mechanical and Aerospace Engineering, Old Dominion University, Norfolk, VA 23529, USA

**Keywords:** electroosmotic flow, viscoelastic fluid, nanofluidics, ionic conductance, electrical double layer

## Abstract

Electroosmotic flow (EOF) of viscoelastic fluid with Linear Phan-Thien–Tanner (LPTT) constitutive model in a nanochannel connecting two reservoirs is numerically studied. For the first time, the influence of viscoelasticity on the EOF and the ionic conductance in the micro-nanofluidic interconnect system, with consideration of the electrical double layers (EDLs), is investigated. Regardless of the bulk salt concentration, significant enhancement of the flow rate is observed for viscoelastic fluid compared to the Newtonian fluid, due to the shear thinning effect. An increase in the ionic conductance of the nanochannel occurs for the viscoelastic fluid. The enhancement of the ionic conductance is significant under the overlapping EDLs condition.

## 1. Introduction

In recent decades, micro/nanofluidics has received significant interest due to its promising applications in bioengineering and chemical engineering [1,2,3,4,5]. Electroosmosis, first reported by Reuss [6], has been widely studied both experimentally and theoretically due to its unique feature of easily manipulating flow at micro/nanoscale [7,8,9,10,11]. At nanoscale, the electric double layer (EDL) may become overlapped under the condition of low bulk salt concentration [12,13], resulting in the ionic selective property of the nanochannel [14].

As is common in chemical and biomedical applications, solutions are often made from large molecules, such as polymer or DNA. These solutions exhibit non-linear rheological behavior that is distinctively different from the Newtonian fluid [15,16], such as the variable viscosity and normal stress difference [17]. Understanding the EOF of these non-Newtonian fluids is of practical importance for the experimental design as well as the operation of various micro/nanofluidic devices. Bello et al. [18] firstly experimentally showed that the electroosmotic flow velocity of a polymer solution in a capillary is much higher than the predicted Helmholtz–Smoluchowski velocity. Chang et al. [19] experimentally investigated the EOF of polymer solutions and observed the drag reduction and reduced effective viscosity. Huang et al. [20] conducted the experimental and theoretical study on the non-Newtonian EOF, and showed the enhancement of the EOF velocity due to the shear thinning effect. Recently, more researches on the EOF of non-Newtonian fluids have been conducted from the theoretical aspect. For example, Zhao et al. [21,22] derived closed-form solutions for the electroosmotic flow of power-law fluids over a planar surface and a parallel-plate microchannel. Tang et al. [23] numerically studied the EOF of power-law non-Newtonian fluid in microchannel, and showed the influence of fluid rheology on the EOF pattern. Choi et al. [24] analytically studied the EOF of viscoelastic fluid with Phan-Thien–Tanner (PTT) model in a two-dimensional microchannel, and analyzed the effects of relaxation time, extensibility parameter, and slip parameter of the PTT model on the velocity and flow rate. Mukherjee et al. [25] developed the closed-form EOF velocity distribution for viscoelastic fluid of the simplified PTT (sPTT) model in microchannel confined between two parallel plates. Martínez et al. [26] asymptotically analyzed the EOF of a viscoelastic fluid with sPTT model in a wavy-wall microchannel, and examined the effects of the wave number and viscoelastic character of the fluid. Park et al. [27] derived the Helmholtz–Smoluchowski velocity and analytically calculated the volumetric flow rate in a microchannel for the EOF of PTT fluid. Afonso et al. [28] developed the analytical solution for the EOF of viscoelastic fluid in a microchannel by using both PTT model and Finitely Extensible Nonlinear Elastic with Peterlin closure (FENE–P) model. Dhinakaran et al. [29] analytically investigated the steady EOF of viscoelastic fluid between parallel plates using the PTT model. 

Most of the theoretical studies on EOF of viscoelastic fluid are in microscale, where the assumptions of small charge density and relatively thin EDL are reasonable. When the characteristic length of the channel is on nanoscale, the EDL thickness becomes comparable to the nanochannel height [30,31], the nonlinear Poisson–Nernst–Planck equations have to be used to solve for the electric potential and ionic concentration. Mei et al. [32] numerically studied the EOF of viscoelastic fluid in a nanoslit and reported the effect of the rheological property of Linear Phan-Thien–Tanner (LPTT) fluid on the fully developed EOF. In this study, the work of Mei et al. [32] is extended to investigate the EOF of the LPTT viscoelastic fluid in a nanochannel connecting two large reservoirs, which is closer to the actual experimental devices. The influence of the rheological property of the viscoelastic fluid on the ionic conductance across the nanochannel is examined with consideration of the EDLs overlapping condition. 

## 2. Mathematical Model

**Figure 1:**
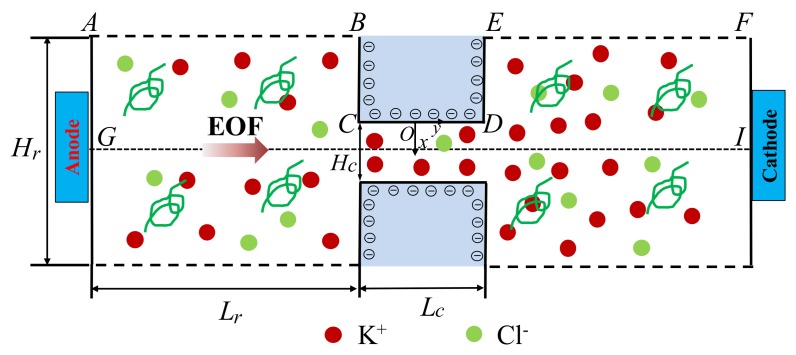
Schematic diagram of a nanochannel connecting two reservoirs at both ends. Uniform negative surface charges are distributed on the nanochannel wall and the adjacent walls of reservoirs. An external electric field is applied by a potential bias between the inlet (Anode) and outlet (Cathode).

Consider a nanochannel of height *H_c_*, length *L_c_*, and width *W* connecting two reservoirs of height *H_r_* and length *L_r_*. A binary KCl electrolyte solution of bulk concentration $C_{0}$ is filled in the nanochannel and is electrically driven by an external potential bias *V*_0_ applied between the inlet (Anode) and outlet (Cathode). Assume that the nanochannel height is much smaller than its width and length (i.e., $H_{c}\ll L_{c},\;H_{c}\ll W$), so the problem can be simplified to a 2D problem schematically shown in Figure 1. Cartesian coordinate system O–xy is adopted with x-axis in the height direction, y-axis in the length direction, and origin fixed on the center of the upper channel surface. As the problem is symmetric about the central axis *GI*, only half of the geometry is considered, with symmetric boundary conditions applied for all fields on the symmetry axis. 

The mass and momentum conservation equations governing the incompressible viscoelastic fluid are

(1) ∇·u=0

(2) $$\rho \left(\frac{\partial u}{\partial t}+u\cdot \nabla u\right)=-\nabla p+2\eta_{s}\nabla \cdot \left[\nabla u+{\left(\nabla u\right)}^{T}\right]+\nabla \cdot \tau -\rho_{e}\nabla \phi$$

where u and *p* denote the velocity field and pressure, respectively; ϕ is the electric potential and $\rho_{e}$ is the volume charge density within the electrolyte solution; *ρ* and $\eta_{s}$ represent the fluid density and the solvent dynamic viscosity, respectively; and the polymeric stress tensor τ accounts for the memory of the viscoelastic fluid. Depending on the type of viscoelastic fluid, different constitutive models have been developed to describe the relation of τ and the deformation rate of the fluid, such as Oldryod–B model, Giesekus model, LPTT model, and so forth. For the LPPT model adopted in this study, τ is given by

(3) $$\tau =\frac{\eta_{p}}{\lambda }\left(c-I\right)$$

where c is the symmetric conformation tensor representing the configuration of the polymer molecules, $\eta_{p}$ is the polymeric viscosity, and λ is the relaxation time of the polymer. 

For the LPTT model, the equation governing the conformation tensor **c** is

(4) $$\frac{\partial c}{\partial t}+u\nabla \cdot c-\left(c\cdot \nabla u^{T}+\nabla u\cdot c\right)=-\frac{1}{\lambda }\left(1+\varepsilon \left(\mathrm{tr}\left(c\right)-3\right)\right)\left(c-I\right)$$

where the non-linear parameter ε is the extensibility parameter. 

The channel surface in contact with the electrolyte solution of permittivity $\varepsilon_{f}$ will become charged and an electric double layer enriched with counterions will develop in the vicinity of the charged surface. The electric potential and ionic concentration within the electrolyte solution are governed by the Poisson equation and the Nernst–Planck equation as

(5) $$-\varepsilon_{f}\nabla^{2}\emptyset =F\left(z_{1}c_{1}^{\prime }+z_{2}c_{2}^{\prime }\right)$$

(6) $$\frac{\partial c_{i}}{\partial t}+\nabla \cdot \left(uc_{i}-D_{i}\nabla c_{i}-z_{i}\frac{D_{i}}{RT}Fc_{i}\nabla \emptyset \right)=0,\;\;\;\;i=1,\;2$$

In the above, $z_{i}$, $D_{i}$ and $c_{i}$ are the valence, diffusivity, and ionic concentration of *i*th ionic species (*i* = 1 for K^+^, 2 for Cl^−^), respectively; F, *R*, and *T* are the Faraday constant, gas constant, and the absolute temperature, respectively. 

Select the channel height *H_c_* as length scale, $U_{0}=\varepsilon_{f}R^{2}T^{2}/\left(\eta_{0}H_{c}F^{2}\right)$ as velocity scale with $\eta_{0}=\eta_{s}+\eta_{p}$ being the total viscosity, $\rho U_{0}{}^{2}$ as the pressure scale, *RT/F* as electric potential scale, the bulk concentration $C_{0}$ as the ionic concentration scale, and the set of governing Equations (1)–(2) and (4)–(6) can be normalized as

(7) $$\nabla^{\prime }\cdot u^{\prime }=0$$

(8) $$\frac{\partial u^{\prime }}{\partial t^{\prime }}+u^{\prime }\cdot \nabla^{\prime }u^{\prime }=-\nabla^{\prime }p^{\prime }+\frac{\beta }{Re}{\nabla^{\prime }}^{2}u^{\prime }+\frac{\left(1-\beta \right)}{Re\;\cdot \;Wi}\nabla^{\prime }\cdot c-\frac{{\left(H_{c}/\lambda_{D}\right)}^{2}}{2Re}\left(z_{1}c_{1}^{\prime }+z_{2}c_{2}^{\prime }\right)\nabla^{\prime }\emptyset^{\prime }$$

(9) $$\frac{\partial c}{\partial t^{\prime }}+u^{\prime }\cdot \nabla^{\prime }c-\left(c\cdot \nabla^{\prime }u^{\prime T}+\nabla^{\prime }u^{\prime }\cdot c\right)=-\frac{1}{Wi}\left(1+\varepsilon \left(\mathrm{tr}\left(c\right)-3\right)\right)\left(c-I\right)$$

(10) $${\nabla^{\prime }}^{2}\emptyset^{\prime }=\frac{1}{2}{\left(\frac{H_{c}}{\lambda_{D}}\right)}^{2}\left(z_{1}c_{1}^{\prime }+z_{2}c_{2}^{\prime }\right)$$

(11) $$\frac{\partial c_{i}^{\prime }}{\partial t^{\prime }}+\nabla^{\prime }\cdot \left(u^{\prime }c_{i}^{\prime }-\frac{D_{i}}{H_{c}U_{0}}\nabla^{\prime }c_{i}^{\prime }-\frac{z_{i}D_{i}}{H_{c}U_{0}}c_{i}^{\prime }\nabla^{\prime }\emptyset^{\prime }\right)=0,i=1,2$$

In the above, all the variables with prime indicate their dimensionless form; the Debye length is $\lambda_{D}=\sqrt{\varepsilon_{f}RT/\sum_{i=1}^{2}F^{2}z_{i}^{2}C_{0}}$; β is the ratio of the solvent viscosity to the total viscosity, i.e., $\beta =\frac{\eta_{s}}{\eta_{0}}$; the dimensionless Reynolds number is $Re=\rho U_{0}H_{c}/\eta_{0}$, and Weissenberg number is $Wi=\lambda U_{0}/H_{c}$. The boundary conditions are given as follows. 

At the symmetric axis, zero normal gradient is applied for all variables. 

At the Anode (or Cathode),

(12) $$n\cdot \nabla^{\prime }u^{\prime }=0,\;\;p^{\prime }=0,\;\emptyset^{\prime }=V_{0}\cdot \frac{F}{RT}\;\left(\mathrm{or}\;0\right),c_{i}^{\prime }=1,\;n\cdot \nabla^{\prime }c=0$$

where n represents the normal unit vector on the surface. 

On the nanochannel wall with a uniform surface charge density $\sigma_{s}$,

(13) $$u^{\prime }=0,\;\;n\cdot \nabla^{\prime }\emptyset^{\prime }=\sigma_{s}\cdot \frac{H_{c}F}{RT},-n\cdot \nabla^{\prime }c_{i}^{\prime }-z_{i}c_{i}^{\prime }n\cdot \nabla^{\prime }\emptyset^{\prime }=0,\;n\cdot \nabla^{\prime }c=0$$

On the surfaces of reservoir (i.e., AB and EF), a symmetric boundary condition is imposed to account for the large size reservoirs. 

The initial conditions are set as

(14) $$u^{\prime }=0,c=I,\;c_{1}^{\prime }=1,\;c_{2}^{\prime }=1,\;\;\emptyset^{\prime }=0,\;\mathrm{at}\;t^{\prime }=0$$

## 3. Numerical Method and Code Validation

One of the most challenging problems for the numerical simulation of viscoelastic fluid flow is the high Weissenberg Number Problem (HWNP), i.e., the loss of numerical accuracy and stability at a relatively high *Wi* [33,34,35]. Log conformation reformulation (LCR) method [34] has been shown as one of the most effective strategy to overcome this issue, and is adopted in this study. The procedure is presented as follows. 

Due to the symmetric positive definite (SPD) property of conformation tensor **c**, its matrix logarithm exists as

(15) $$\Psi =\log\left(c\right)=R^{T}\log\left(\Lambda \right)R$$

where Λ is a diagonal matrix whose diagonal elements are the eigenvalues of ***c***; and ***R*** is an orthogonal matrix composed of the eigenvectors of ***c***.

The equation for the conformation tensor can be rewritten in terms of Ψ as

(16) $$u^{\prime }\cdot \nabla^{\prime }\Psi -\left(\Omega \cdot \Psi -\Psi \cdot \Omega \right)-2B=-\frac{1}{Wi}e^{-\Psi }\left(1+\varepsilon \left(\mathrm{tr}\left(e^{\;\Psi }\right)-3\right)\right)\left(e^{\;\Psi }-I\right)$$

where Ω and B are the anti-symmetric matrix and the symmetric traceless matrix of the decomposition of the velocity gradient tensor $\nabla^{\prime }u^{\prime }$, as derived by Fattal and Kupferman [35]. 

Then, the conformation tensor **c** can be obtained from Ψ as

(17) c=exp(Ψ)

To numerically solve the coupled set of Equations (7)–(8), (10)–(11), and (16) along with the boundary and initial conditions, a new solver is implemented in an open source software for CFD–OpenFOAM. QUICK, Gauss Linear, and MINMOD schemes are used to discretize the convection terms in Equations (8), (11), and (16), respectively. Pressure Implicit with Splitting of Operators (PISO) algorithm is used to solve Equation (8). Finer mesh is distributed near the charged wall and a mesh convergence study is conducted to ensure the accuracy of the following simulations. 

The developed solver has been shown to accurately simulate the viscoelastic fluid of EOF in a nanoslit [32]. To further check the accuracy and the validity of the developed solver, we simulate a Newtonian EOF in a nanochannel with reservoirs, the geometry of which is used for the following study, and compare the results with that obtained from finite element software Comsol (version 5.1, Comsol, Stockholm, Sweden). The geometric parameters are given as $H_{c}=20\;\mathrm{nm},\;L_{c}=100\;\mathrm{nm},H_{r}=200\;\mathrm{nm},\;\;L_{r}=200\;\mathrm{nm},\;W=1\;\mu m.$ Other parameters are set as $V_{0}=0.05\;V,\;\sigma_{s}=-0.005\;c/m^{2},\;\;D_{1}\left(D_{2}\right)=1.96\;\left(2.03\right)\times 10^{-9}m^{2}s^{-1},\;\varepsilon_{f}=7.08\times 10^{-10}{\mathrm{CV}}^{-1}m^{-1}\;$. For the simulations in this study, the time is set long enough that all flows reach steady state, and the steady results are shown below.

**Figure 2:**
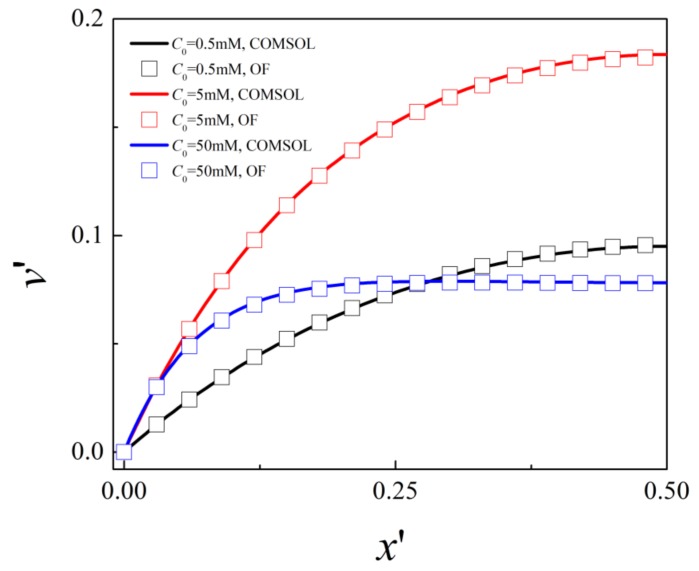
Distribution of the dimensionless axial velocity at the center of the nanochannel for bulk salt concentrations *C*_0_ = 0.5 mM, 5 mM, and 50 mM: symbols (OpenFOAM) and lines (Comsol).

Figure 2 shows the comparison of the simulated dimensionless velocity in the channel length direction at the middle cross section of the nanochannel with the results obtained in commercial software Comsol (www.comsol.com) for different bulk salt concentrations, *C*_0_ = 0.5, 5, and 50 mM, corresponding to $\frac{H_{c}}{2\lambda_{D}}=0.74,\;2.33,\;\mathrm{and}\;7.35$. Under low salt concentration *C*_0_ = 0.5 mM, as the thickness of EDL is larger than half of the channel height, i.e., the EDLs are overlapping, the velocity gradually increases from the wall and reaches maximum velocity at the center of the nanochannel. For high bulk salt concentration *C*_0_ = 50 mM with thin EDL, the velocity increases to its maximum value within a distance from the charged surface and remains at its maximum value. It is obvious that good agreement between our numerical results and the Comsol simulation is achieved for both cases of thin EDL and overlapping EDLs. 

The EOF of viscoelastic fluid in the same geometry is then solved by the validated solver to investigate the effects of Weissenberg number *Wi* on the flow rate and ionic conductance. The viscosity ratio and the extensibility parameter for the LPTT viscoelastic fluid are set to β = 0.1 and ε = 0.25. 

## 4. Results and Discussion

**Figure 3:**
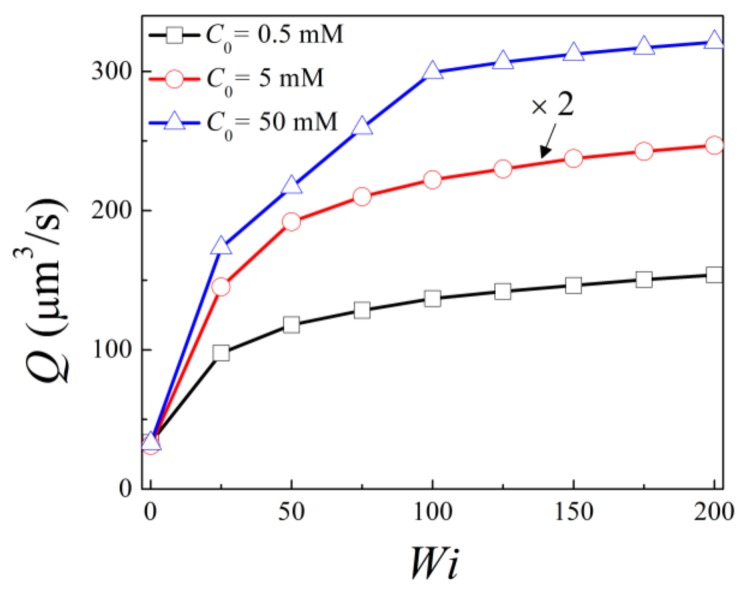
Variation of volume flow rate with the Weissenberg number for bulk concentration *C*_0_ = 0.5 mM, 5 mM, and 50 mM.

First of all, the volume flow rate across the channel is calculated at the middle of the channel ($y^{\prime }=0$) as $Q=2WH_{c}U_{0}\int_{0}^{1/2}v^{\prime }dx^{\prime }$ for bulk salt concentration *C*_0_ = 0.5 mM, 5 mM, and 50 mM under different Weissenberg *Wi*, as shown in Figure 3. With the same Weissenberg number, the volume flow rate is lowest at C_0_ = 0.5 mM, while it is the highest at C_0_ = 5 mM. The former is because for low bulk salt concentration C_0_ = 0.5 mM, the overall net charge density and thus the electric body force within the nanochannel is low. The highest flow rate at moderate bulk salt concentration C_0_ = 5 mM is due to the fact that the EDLs are slightly overlapped, so the net ionic concentration within the nanochannel is higher compared to those for both C_0_ = 0.5 mM and C_0_ = 50 mM. Under the same bulk salt concentrations, Q monotonously increases with *Wi* within the investigated range, which is due to the shear thinning effect of viscoelastic fluid of the LPTT model. Besides this, the increase of flow rate is more obvious for Wi<50 and becomes less apparent as Wi further increases. This indicates that the effect of *Wi* on the shear viscosity becomes less apparent with increasing *Wi*. At *Wi* =200, the flow rates are 4.95, 7.89, and 9.74 times of that for Newtonian fluid at *C*_0_ = 0.5 mM, 5 mM, and 50 mM, respectively. This indicates that the shear thinning effect is more obvious for the case with smaller EDL thickness. 

The ionic conductance within the nanochannel is calculated as

(18) $$G=\frac{I}{V_{0}}=\frac{2W}{V_{0}}\int_{0}^{H_{r}/2}\overset{2}{\underset{i=1}{\sum }}Fz_{i}\left(vc_{i}-D_{i}\frac{\partial c_{i}}{\partial y}-z_{i}\frac{D_{i}}{RT}Fc_{i}\frac{\partial \emptyset }{\partial y}\right)dx$$

In terms of dimensionless variables, the ionic conductance can be written as

(19) $$\begin{array}{l} G=\frac{2C_{0}\varepsilon_{f}R^{2}T^{2}W}{V_{0}\eta_{0}\text{F}}\int_{0}^{\frac{H_{r}}{2H_{c}}}\overset{2}{\underset{i=1}{\sum }}z_{\text{i}}\left(vc_{i}^{\prime }-\frac{D_{i}}{H_{c}U_{0}}\frac{\partial c_{i}^{\prime }}{\partial y^{\prime }}-\frac{z_{i}D_{i}}{H_{c}U_{0}}c_{i}^{\prime }\frac{\partial \emptyset^{\prime }}{\partial y^{\prime }}\right)dx^{\prime }\\ \;\;\;=\frac{2C_{0}\varepsilon_{f}R^{2}T^{2}W}{V_{0}\eta_{0}\text{F}}\int_{0}^{\frac{H_{r}}{2H_{c}}}(v^{\prime }c_{1}^{\prime }-\frac{D_{1}}{H_{c}U_{0}}\frac{\partial c_{1}^{\prime }}{\partial y^{\prime }}-\frac{D_{1}}{H_{c}U_{0}}c_{1}^{\prime }\frac{\partial \emptyset^{\prime }}{\partial y^{\prime }})\\ \;\;\;-(v^{\prime }c_{2}^{\prime }-\frac{D_{2}}{H_{c}U_{0}}\frac{\partial c_{2}^{\prime }}{\partial y^{\prime }}+\frac{D_{2}}{H_{c}U_{0}}c_{2}^{\prime }\frac{\partial \emptyset^{\prime }}{\partial y^{\prime }})dx^{\prime } \end{array}$$

It is obvious that the ionic conductance consists of convective, diffusive, and migrative components, which can be written as

(20a) $$G_{c}=\frac{2C_{0}\varepsilon_{f}R^{2}T^{2}W}{V_{0}\eta_{0}F}\int_{0}^{\frac{H_{r}}{2H_{c}}}v^{\prime }\left(c_{1}^{\prime }-c_{2}^{\prime }\right)dx^{\prime }$$

(20b) $$G_{d}=\frac{2C_{0}\varepsilon_{f}R^{2}T^{2}W}{V_{0}\eta_{0}F}\int_{0}^{\frac{H_{r}}{2H_{c}}}\frac{1}{H_{c}U_{0}}\left(-D_{1}\frac{\partial c_{1}^{\prime }}{\partial y^{\prime }}+D_{2}\frac{\partial c_{2}^{\prime }}{\partial y^{\prime }}\right)dx^{\prime }$$

(20c) $$G_{m}=\frac{2C_{0}\varepsilon_{f}R^{2}T^{2}W}{V_{0}\eta_{0}F}\int_{0}^{\frac{H_{r}}{2H_{c}}}\frac{1}{H_{c}U_{0}}\frac{\partial \emptyset^{\prime }}{\partial y^{\prime }}\left(-D_{1}c_{1}^{\prime }-D_{2}c_{2}^{\prime }\right)dx^{\prime }$$

**Figure 4:**
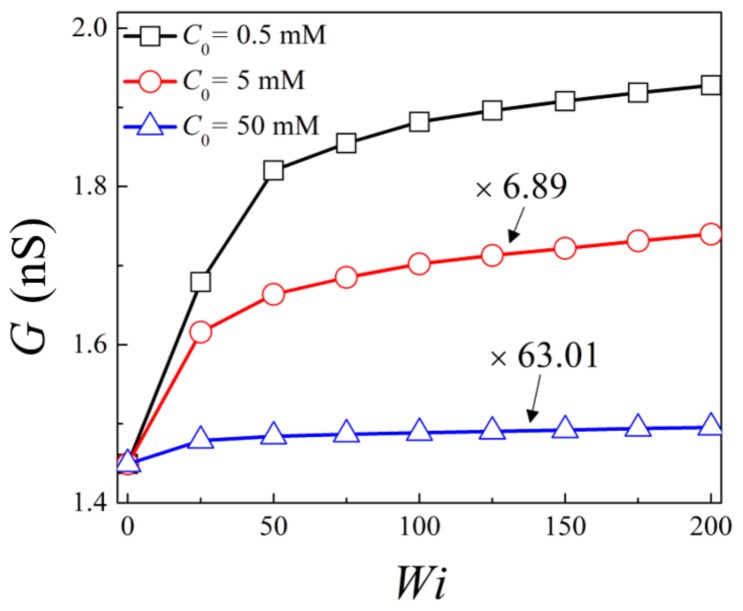
Variation of ionic conductance with Weissenberg number for *C*_0_ = 0.5 mM, 5 mM, and 50 mM.

Figure 4 shows the variation of the ionic conductance with the Weissenberg number for bulk salt concentrations of *C*_0_ = 0.5 mM, 5 mM, and 50 mM, respectively. For *C*_0_ = 0.5 mM and 5 mM, apparent increase of ionic conductance is seen for viscoelastic fluid compared to that of the Newtonian fluid, and the ionic conductance monotonously increase with *Wi*. Similar to the trend of the flow rate, the increase becomes less obvious for higher *Wi*. For 50 mM, ionic conductance slightly increases for viscoelastic fluid at *Wi* = 50 compared to the Newtonian fluid, and remains almost constant as *Wi* further increases. At *Wi* = 200, the ionic conductance is 1.27, 1.20, and 1.03 times that for Newtonian fluid under bulk salt concentrations of *C*_0_ = 0.5 mM, 5 mM, and 50 mM, respectively. Thus, the enhancement of ionic conductance is more obvious for low salt concentration, under which the EDLs are highly overlapped, and becomes less apparent as *C*_0_ increases. When the bulk salt concentration is relatively high, the effect of viscoelasticity on the ionic conductance is negligible. 

**Figure 5:**
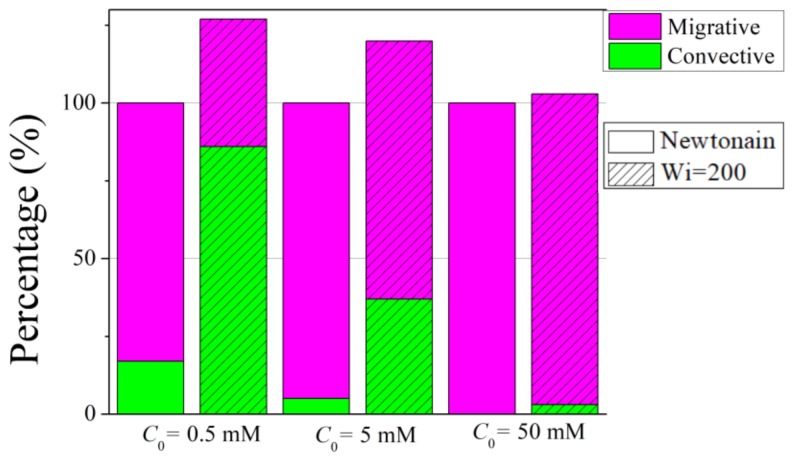
Percentage of the convective and migrative ionic conductance components for Newtonian and viscoelastic fluids of *Wi* = 200 for C_0_ = 0.5 mM, 5 mM, and 50 mM, respectively. For clarity, the percentage of the components for viscoelastic fluid is shown with respect to the Newtonian fluid under the same bulk salt concentration. Thus, the total height for viscoelastic fluid becomes 127%, 120%, and 103% for C_0_ = 0.5 mM, 5 mM, and 50 mM, respectively.

The variation of ionic conductance with *Wi* for different bulk concentrations can be analyzed by the contributions of the convective, diffusive, and migrative components in Equation (20). Figure 5 presents the percentage of the convective and migrative components for Newtonian fluid and viscoelastic fluid of *Wi* = 200 at C_0_ = 0.5 mM, 5 mM and 50 mM, respectively. The diffusive component is not shown due to the fact that for all cases, the percentage of diffusive conductance is less than 0.5%, and thus its contribution is negligible. Besides this, to better compare the results of the Newtonian fluid and the viscoelastic fluid, the percentage of viscoelastic is shown with respect to the Newtonian fluid under the same C_0_. For viscoelastic of *Wi* = 200, an apparent increase in the convective ionic conductance is observed compared to that of the Newtonian fluid, while an obvious decrease of migrative component is seen for viscoelastic fluid. The increase of the convective ionic conductance is due to shear thinning effect, as it is proportional to the mainstream velocity. The decrease of migrative ionic conductance stems from the change in the distributions of the electric potential and the ionic concentration under the presence of viscoelasticity. The increase of the convective component exceeds the decrease of the migrative component, resulting in an overall increase of the ionic conductance. Besides this, at low bulk salt concentration, the ratio of the convective component to the migrative component is relatively large, thus the increase of convective component for viscoelastic fluid contributes significantly to the increase of the total ionic conductance. As the bulk salt concentration increases, the contribution of the convective component becomes smaller, thus the increase of the total ionic conductance for viscoelastic fluid becomes less significant. In Newtonian fluid, the migrative component dominates for all three salt concentrations, while in viscoelastic fluid the convective component dominates when the EDLs are overlapped. 

**Figure 6:**
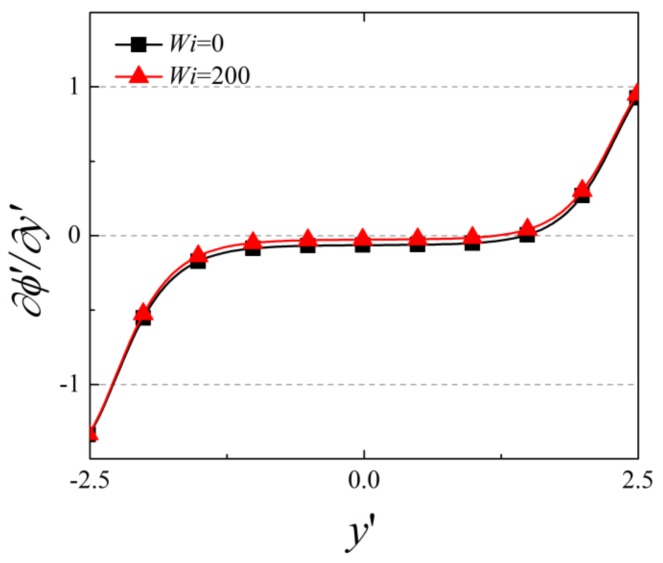
The variation of $\frac{\partial \emptyset^{\prime }}{\partial y^{\prime }}$ along the symmetry axis of the nanochannel for C_0_ = 0.5 mM.

Figure 6 depicts the distribution of the dimensionless $\frac{\partial \emptyset^{\prime }}{\partial y^{\prime }}$ along the centerline of the nanochannel for Newtonian fluid and viscoelastic fluid of *Wi* = 200 for the case of overlapped EDLs (i.e., C_0_ = 0.5 mM). Within the confined nanochannel, the value of $\frac{\partial \emptyset^{\prime }}{\partial y^{\prime }}$ remains almost constant, while a sharp increase (decrease) occurs near the opening at the Cathode (Anode) side. This variation arises from the large change of net charge density within the solution in both reservoirs compared to the region near the nanochannel, due to the highly overlapped EDLs. Besides this, it is noticed that within the confined nanochannel, a decrease in the magnitude of $\frac{\partial \emptyset^{\prime }}{\partial y^{\prime }}$ occurs for viscoelastic fluid compared to the Newtonian fluid. This decrease explains the decrease in the migrative ionic conductance, which is proportional to $\frac{\partial \emptyset^{\prime }}{\partial y^{\prime }}$. However, the effect of viscoelasticity on the electric potential distribution is not significant. This can be explained as following. As the surface charge density is assumed to be uniformly distributed at the nanochannel wall, the induced EOF velocity is almost parallel to the nanochannel surface. Under this condition, the ionic distribution and thus the electric potential is mainly determined by the surface charge density, and the increase of EOF velocity has negligible effect on the electric potential [36]. 

## 5. Conclusions

Numerical simulation on the EOF of viscoelastic fluid with an LPTT model in a nanochannel connecting two reservoirs is carried out with a new finite volume solver implemented in OpenFOAM. The implemented solver is validated by comparing the result of the current simulation with that from commercial finite element software Comsol for Newtonian fluid with the same geometry. For the first time, the condition of highly overlapped EDLs is taken into consideration for the EOF of viscoelastic fluid. Besides this, the surface charge density is much larger than the typical value used for the theoretical study in microscale, where the linearization is used to solve for the electric potential. Obvious increase in the volume flow rate is obtained for the viscoelastic fluid compared to Newtonian fluid due to the shear thinning effect of LPTT fluid. The enhancement is more significant under high bulk salt concentration where EDL is not overlapped. An enhancement in ionic conductance also occurs for viscoelastic fluid, and the enhancement becomes less significant as the bulk salt concentration increases. The increase of the ionic conductance arises from the increase of its convective component, which is directly proportional to the enhanced EOF velocity. In contrast to Newtonian fluid, where migrative ionic conductance always dominates over the convective and diffusive components, the convective current becomes dominant when the EDLs are overlapped for viscoelastic fluid. As the bulk salt concentration increases, the contribution of the convective ionic conductance decreases and the enhancement in the ionic conductance for the viscoelastic fluid becomes less significant. 

## References

[1] Hsu W.-L., Daiguji H. (2016). Manipulation of protein translocation through nanopores by flow field control and application to nanopore sensors. Anal. Chem..

[2] Park M.C., Kim M., Lim G.T., Kang S.M., An S.S.A., Kim T.S., Kang J.Y. (2016). Droplet-based magnetic bead immunoassay using microchannel-connected multiwell plates (μCHAMPs) for the detection of amyloid beta oligomers. Lab Chip.

[3] Chen X., Zhang S., Zhang L., Yao Z., Chen X., Zheng Y., Liu Y. (2017). Applications and theory of electrokinetic enrichment in micro-nanofluidic chips. Biomed. Microdevices.

[4] Plecis A., Nanteuil C.M., Haghiri-Gosnet A.-M., Chen Y. (2008). Electropreconcentration with charge-selective nanochannels. Anal. Chem..

[5] Karniadakis G., Beskok A., Aluru N. (2006). Microflows and Nanoflows: Fundamentals and Simulation.

[6] Reuss F. (1809). Charge-induced flow. Proc. Imp. Soc. Nat. Mosc..

[7] Li L., Wang X., Pu Q., Liu S. (2019). Advancement of electroosmotic pump in microflow analysis: A review. Anal. Chimica Acta.

[8] Di Fraia S., Massarotti N., Nithiarasu P. (2018). Modelling electro-osmotic flow in porous media: A review. Int. J. Numer. Method. Heat Fluid Flow.

[9] Shehzad N., Zeeshan A., Ellahi R. (2018). Electroosmotic flow of MHD power law Al2O3-PVC nanouid in a horizontal channel: Couette-Poiseuille flow model. Commun. Theor. Phys..

[10] Ma Y., Yeh L.-H., Lin C.-Y., Mei L., Qian S. (2015). pH-regulated ionic conductance in a nanochannel with overlapped electric double layers. Anal. Chem..

[11] Rashidi S., Bafekr H., Valipour M.S., Esfahani J.A. (2018). A review on the application, simulation, and experiment of the electrokinetic mixers. Chem. Eng. Proc. Process Intensif..

[12] Huang M.-J., Mei L., Yeh L.-H., Qian S. (2015). pH-Regulated nanopore conductance with overlapped electric double layers. Electrochem. Commun..

[13] Baldessari F. (2008). Electrokinetics in nanochannels: Part I. Electric double layer overlap and channel-to-well equilibrium. J. Colloid Interface Sci..

[14] Kim S.J., Wang Y.-C., Lee J.H., Jang H., Han J. (2007). Concentration polarization and nonlinear electrokinetic flow near a nanofluidic channel. Phys. Rev. Lett..

[15] Zimmerman W., Rees J., Craven T. (2006). Rheometry of non-Newtonian electrokinetic flow in a microchannel T-junction. Microfluid. Nanofluid..

[16] Nam J., Lim H., Kim D., Jung H., Shin S. (2012). Continuous separation of microparticles in a microfluidic channel via the elasto-inertial effect of non-Newtonian fluid. Lab Chip.

[17] Zhao C., Yang C. (2011). Electro-osmotic mobility of non-Newtonian fluids. Biomicrofluidics.

[18] Bello M.S., De Besi P., Rezzonico R., Righetti P.G., Casiraghi E. (1994). Electroosmosis of polymer solutions in fused silica capillaries. Electrophoresis.

[19] Chang F.-M., Tsao H.-K. (2007). Drag reduction in electro-osmosis of polymer solutions. Appl. Phys. Lett..

[20] Huang Y., Chen J., Wong T., Liow J.-L. (2016). Experimental and theoretical investigations of non-Newtonian electro-osmotic driven flow in rectangular microchannels. Soft Matter.

[21] Zhao C., Yang C. (2011). An exact solution for electroosmosis of non-Newtonian fluids in microchannels. J. Non-Newton. Fluid Mech..

[22] Zhao C., Yang C. (2010). Nonlinear Smoluchowski velocity for electroosmosis of Power-law fluids over a surface with arbitrary zeta potentials. Electrophoresis.

[23] Tang G., Li X., He Y., Tao W. (2009). Electroosmotic flow of non-Newtonian fluid in microchannels. J. Non-Newton. Fluid Mech..

[24] Choi W., Joo S.W., Lim G. (2012). Electroosmotic flows of viscoelastic fluids with asymmetric electrochemical boundary conditions. J. Non-Newton. Fluid Mech..

[25] Mukherjee S., Das S.S., Dhar J., Chakraborty S., DasGupta S. (2017). Electroosmosis of viscoelastic fluids: Role of wall depletion layer. Langmuir.

[26] Martínez L., Bautista O., Escandón J., Méndez F. (2016). Electroosmotic flow of a Phan-Thien–Tanner fluid in a wavy-wall microchannel. Colloid. Surf. A Physicochem. Eng. Asp..

[27] Park H., Lee W. (2008). Helmholtz–Smoluchowski velocity for viscoelastic electroosmotic flows. J. Colloid Interface Sci..

[28] Afonso A., Alves M., Pinho F. (2009). Analytical solution of mixed electro-osmotic/pressure driven flows of viscoelastic fluids in microchannels. J. Non-Newton. Fluid Mech..

[29] Dhinakaran S., Afonso A., Alves M., Pinho F. (2010). Steady viscoelastic fluid flow between parallel plates under electro-osmotic forces: Phan-Thien–Tanner model. J. Colloid Interface Sci..

[30] Mei L., Yeh L.-H., Qian S. (2016). Gate modulation of proton transport in a nanopore. Phys. Chem. Chem. Phys..

[31] Haywood D.G., Harms Z.D., Jacobson S.C. (2014). Electroosmotic flow in nanofluidic channels. Anal. Chem..

[32] Mei L., Zhang H., Meng H., Qian S. (2018). Electroosmotic flow of viscoelastic fluid in a nanoslit. Micromachines.

[33] Comminal R., Spangenberg J., Hattel J.H. (2015). Robust simulations of viscoelastic flows at high Weissenberg numbers with the streamfunction/log-conformation formulation. J. Non-Newton. Fluid Mech..

[34] Fattal R., Kupferman R. (2005). Time-dependent simulation of viscoelastic flows at high Weissenberg number using the log-conformation representation. J. Non-Newton. Fluid Mech..

[35] Fattal R., Kupferman R. (2004). Constitutive laws for the matrix-logarithm of the conformation tensor. J. Non-Newton. Fluid Mech..

[36] Grigoriev R., Schuster H.-G. (2012). Transport and Mixing in Laminar Flows: From Microfluidics to Oceanic Currents.

